# Iron plaques as terminal electron acceptors optimize clostridial fermentation and nitrogen fixation in rice rhizospheres

**DOI:** 10.1093/ismejo/wrag088

**Published:** 2026-04-14

**Authors:** Ji Zhan, Wei Yang, Junhui Guo, Yanshuang Yu, Shuyao Lai, Xing Liu, Shungui Zhou

**Affiliations:** Fujian Provincial Key Laboratory of Soil Environmental Health and Regulation, College of Resources and Environment, Fujian Agriculture and Forestry University, Fuzhou 350002, China; Fujian Provincial Key Laboratory of Soil Environmental Health and Regulation, College of Resources and Environment, Fujian Agriculture and Forestry University, Fuzhou 350002, China; Fujian Provincial Key Laboratory of Soil Environmental Health and Regulation, College of Resources and Environment, Fujian Agriculture and Forestry University, Fuzhou 350002, China; Institute of Resources, Environment and Soil Fertilizer, Fujian Academy of Agricultural Sciences, Fuzhou 350003, China; Fujian Provincial Key Laboratory of Soil Environmental Health and Regulation, College of Resources and Environment, Fujian Agriculture and Forestry University, Fuzhou 350002, China; Fujian Provincial Key Laboratory of Soil Environmental Health and Regulation, College of Resources and Environment, Fujian Agriculture and Forestry University, Fuzhou 350002, China; Fujian Provincial Key Laboratory of Soil Environmental Health and Regulation, College of Resources and Environment, Fujian Agriculture and Forestry University, Fuzhou 350002, China

**Keywords:** nitrogen conversion, paddy soil, Gram-positive microorganisms, extracellular electron transfer, fermentative iron reduction, fermentation shift, intracellular redox balance

## Abstract

Fermentative *Clostridium* species associated with rice roots can contribute substantially to biological nitrogen fixation (BNF) in anoxic paddy soils, yet whether their BNF is regulated by the redox chemistry of rhizosphere remains unclear. Here, we show that iron plaques on rice roots function as terminal electron acceptors that reprogram *Clostridium* fermentation and thereby enhance BNF. In nitrogen-fixation microcosms, *Clostridium sensu stricto I* was selectively enriched under plaque-associated Fe(III)-reducing conditions, coinciding with elevated nitrogen fixation. Metabolomic profiling coupled with metabolic flux analysis revealed that Fe(III) reduction redirects a portion of carbon and electron flow from low-energy-yield solventogenesis toward high-energy-yield acidogenesis. This shift increases cellular ATP generation and expands the reductant pool, thereby benefiting the energetic and reductant demands of nitrogenase. Integrated transcriptomic and metagenomic analyses further identified NosR, a flavin mononucleotide-binding protein that is upregulated during Fe(III) reduction and may facilitate electron delivery to plaque-associated Fe(III). Our findings establish a mechanism in which iron plaque reduction optimizes fermentation for BNF, providing fundamental insights into coupled Fe–N cycling in rice rhizospheres and suggesting potential strategies for sustainable nitrogen management in flooded agroecosystems.

## Introduction

Paddy soils are a distinctive agroecosystem that underpins food security for a large fraction of the global population [1, 2]. Sustaining their productivity depends strongly on maintaining adequate nitrogen (N) supply [3]. Although synthetic N fertilizers are the dominant input for achieving high yields, non-symbiotic biological nitrogen fixation (BNF) by soil microorganisms is a major natural source of reactive N and contributes to the long-term fertility of paddy soils [4, 5]. Estimates suggest that non-symbiotic BNF can contribute on the order of ~22 kg N ha^−1^ year^−1^ to rice systems, and, under some conditions, can help sustain moderate yields in the absence of mineral N fertilizer [6, 7]. This potential stems from the biochemistry of nitrogenase, the enzyme responsible for BNF, which is irreversibly damaged by oxygen and demands substantial ATP and electrons [1, 8]. The prolonged flooding of paddy soils creates anoxic, carbon-rich conditions that are ideally suited to protect nitrogenase from oxygen and meet the high energy demands of BNF, making these environments particularly favorable for non-symbiotic nitrogen fixation compared with well-aerated upland soils [9].

**Figure 1:**
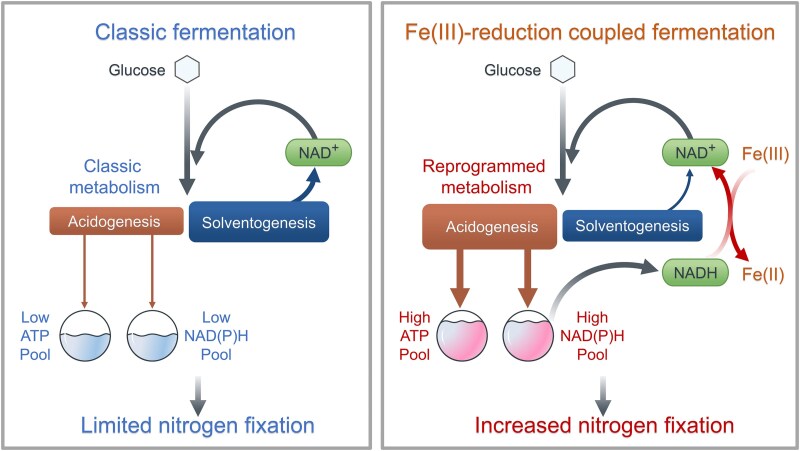
Fermentative iron plaque reduction enhances nitrogen fixation in *Clostridium*. In classic fermentation, glucose is metabolized via a balanced flux between acidogenesis (generating ATP and NAD(P)H) and solventogenesis (regenerating NAD^+^ to maintain redox homeostasis). In the presence of iron plaque, extracellular Fe(III) reduction serves as an alternative electron sink, efficiently regenerating NAD^+^. This relieves intracellular redox pressure, reprogramming metabolism by shifting the carbon flux from solventogenesis toward acidogenesis. The resulting increase in ATP and NAD(P)H pools ultimately supports enhanced nitrogen fixation.

A defining feature of flooded paddy soils is their low redox potential, which is tightly coupled to iron redox cycling [10, 11]. Alternating flooding and drainage promote Fe(II) oxidation during oxic periods and microbial Fe(III) reduction during anoxic periods, sustaining dynamic Fe(III)/Fe(II) transformations [12]. Iron cycling can influence non-symbiotic BNF not only indirectly by shaping redox conditions, but also directly by supplying bioenergetic opportunities [13]. Several extracellular Fe(III)-reducing bacteria, including genera such as *Geobacter* and *Anaeromyxobacter*, are also diazotrophs; they conserve energy through Fe(III) respiration and can channel that energy toward nitrogenase activity [14–16]. Consequently, Fe(III)-respiring diazotrophs often dominate the nitrogen-fixing microbial community in paddy fields and Fe(III) respiration represents a major contribution to soil nitrogen fixation [13, 14]. These observations suggest a broader view that the availability and dynamics of external Fe(III) can structure nitrogen-fixing communities and modulate nitrogen input rates in anoxic soils. In parallel, paddy soils also harbor abundant fermentative diazotrophs, particularly members of the genus *Clostridium*, which have repeatedly been implicated as significant contributors to non-symbiotic BNF in flooded systems [17, 18].

*Clostridium* spp. typically fuel BNF by fermenting organic carbon [19, 20], but this process imposes a fundamental constraint: energy conservation and redox balancing are coupled in ways that can limit simultaneous availability of ATP and reductant for nitrogenase [21–23]. During acidogenesis, fermentative carbon is routed mainly to organic acids (e.g. acetate and butyrate). This acid-forming metabolism couples carbon flux to substrate-level phosphorylation, generating ATP while producing reduced equivalents such as NADH and reduced ferredoxin [24]. As these reduced carriers build up, cells shift toward solventogenesis, in which acids are reassimilated and reducing equivalents are consumed to produce more reduced products (e.g. butanol, ethanol, and acetone) and other reductant-consuming pathways to maintain redox balance. Because solventogenesis channels carbon into products without the same ATP-generating substrate-level phosphorylation steps, it generally yields less net ATP than acidogenesis [24]. This trade-off can restrict how efficiently diazotrophic *Clostridium* spp. meet the high ATP and electron demand of nitrogen fixation. However, several *Clostridium* species such as *Clostridium acetobutylicum* [25], *Clostridium pasteurianum* [26], *Clostridium butyricum*  [27], and *Clostridium saccharobutylicum* [28] can reduce Fe(III) during fermentation. By exporting reducing equivalents to Fe(III) as an external sink, these organisms modulate their intracellular redox balance, thereby shifting their fermentation metabolism [29]. This raises a key unresolved question for paddy soils: **can Fe(III) redox dynamics regulate clostridial fermentation in ways that enhance their contribution to BNF?**

The rice rhizosphere couples high organic carbon availability (root exudation) with steep redox gradients and rapid Fe(II)–Fe(III) turnover at fine spatial scales [30–32]. Oxygen release from roots promotes Fe(II) oxidation and the formation of iron plaques on root surfaces [32, 33], creating a reactive Fe(III) reservoir directly adjacent to microbial communities that experience anoxia and abundant substrates [34, 35]. Despite extensive work on iron plaque formation and its biogeochemical effects, whether rhizosphere iron plaques serve as terminal electron acceptors that reorganize fermentative metabolism, and thereby regulate BNF, remains unclear.

In this study, we employed an integrated approach, combining nitrogen-fixation microcosms, strain isolation, and a multi-omics framework (metabolomics, metabolic flux analysis, transcriptomics, and metagenomics), to investigate the impact of rhizosphere iron plaques on fermentative diazotrophs. Our results show that iron plaques act as biogeochemical selectors that enrich specific *Clostridium* lineages and that plaque-associated Fe(III) reduction enhances BNF. Mechanistically, Fe(III) reduction relieves intracellular redox pressure, inducing a partial shift in metabolic flux from solventogenesis toward high-yield acidogenesis. This increases ATP synthesis and expands the reducing equivalent pool available to nitrogenase (Fig. 1). We further identify a candidate extracellular electron transfer (EET) module involving flavinylated NosR that may facilitate electron delivery to iron plaque. Together, our findings establish a mechanistic connection between rhizosphere iron plaque redox chemistry, fermentative metabolism, and microbial nitrogen fixation, with implications for coupled Fe–N cycling and sustainable nitrogen management in flooded agroecosystems.

## Materials and methods

### Iron plaque induction and microcosm setup

Rice (*Oryza sativa* L.) seedlings were surface-sterilized and cultivated in International Rice Research Institute (IRRI) nutrient solution until 30 days old, as described previously [36]. The roots of 30-day-old seedlings were then immersed in deionized water for 24 h. Subsequently, uniformly grown seedlings were treated with 100 mg l^−1^ FeSO_4_·7H_2_O for 3 days to induce iron plaque formation, whereas a control group was treated with deionized water without FeSO_4_·7H_2_O. Successful iron plaque formation was confirmed by the appearance of a distinct reddish-brown coating on the root surface and by Raman spectroscopy showing characteristic signatures consistent with ferrihydrite (Supplementary Fig. S1a).

To construct rice rhizosphere microcosms, 1.2 g of fresh roots, with or without iron plaque coating, were transferred into anoxic bottles containing 20 ml of modified N-free Hoagland solution (pH 6.0). Nitrogen was omitted by replacing all N-containing salts (Ca(NO₃)₂·4H₂O, KNO₃, and NH₄H₂PO₄) with equimolar amounts of their chloride- or sulfate-based counterparts (CaCl₂·2H₂O, KH₂PO₄, and K₂SO₄). The root-solution system was deoxygenated with N_2_, sealed with a rubber stopper, and sterilized by autoclaving at 121°C for 20 min. Afterward, filter-sterilized glucose was added to each microcosm to achieve a final concentration of 20 mM, simulating root exudates [30]. The microbial inoculum was prepared by resuspending 1 g of rice rhizosphere soil collected from experimental fields at Fujian Agriculture and Forestry University (26°05′2.90″N, 119°14′3.66″E) in 50 ml of sterile deionized water. An aliquot (100 μl) of the resulting suspension was then collected and used as the inoculum. A summary of physicochemical properties of the soil were provided in Supplementary Table S1. All incubations were performed in triplicate at 30°C under dark conditions.

### Nitrogen fixation assays

Whole microcosm or pure cell cultures were lysed by sonication on ice using an ultrasonic homogenizer (JY92-IIDN, Ningbo Scientz Biotechnology Co., Ningbo, China) at 200 W with 1 s on/1 s off pulses for 10 min. The lysates were then analyzed for total nitrogen (*N*_total_) using the persulfate digestion method (Hach kits 2 672 245, USA) following the manufacturer’s instructions [37]. Nitrogen fixation was quantified as the net increase in *N*_total_ over the incubation period.

Nitrogenase activity was assessed via the acetylene (C_2_H_2_) reduction assay [38] Briefly, when cultures in the microcosm reached mid-log phase, the headspace was purged with an Ar–C_2_H_2_ mixture (90:10, v/v) for 30 min. After 24 h of incubation, 0.3 ml of headspace gas was sampled and analyzed for ethylene (C_2_H_4_) production from C_2_H_2_ using a gas chromatography system equipped with a flame ionization detector (GC-FID; GC-2014, Shimadzu, Japan). Separation was achieved using a Porapak N 50-80 mesh capillary column (4 m × 4 mm i.d.). Nitrogenase activity was expressed as micromoles of ethylene produced per hour per milligram of protein (nmol C_2_H_4_ h^−1^ mg^−1^ protein), with protein concentration determined using a MicroBCA Protein Assay Kit (Thermo Fisher Scientific, Rockford, USA).

For ^15^N_2_ isotope labeling, 10% (v/v) of microcosm’s headspace gas was replaced with 99.9% pure ^15^N_2_ (Aladdin, Shanghai, China). After incubation, microbial biomass was harvested by centrifugation (8000 × g, 10 min, 4°C). The resulting pellet was lyophilized, and its ^15^N enrichment was analyzed using an elemental analyzer coupled to an isotope ratio mass spectrometer (EA-IRMS; Integra2, Sercon, UK).

### Microbial community analysis

Genomic DNA was extracted from rhizosphere microcosm samples using the FastDNA Spin Kit for Soil (MP Biomedicals, Santa Ana, CA, USA) following the manufacturer’s instructions. The hypervariable V3-V4 region of the bacterial 16S rRNA gene was amplified using the universal primers 338F (5′-ACTCCTACGGGAGGCAGCAG-3′) and 806R (5′-GGACTACHVGGGTWTCTAAT-3′). The amplicons were purified, pooled in equimolar amounts, and subjected to paired-end sequencing on a NextSeq 2000 System (Illumina, San Diego, CA, USA) by Majorbio Bio-Pharm Technology Co., Ltd. (Shanghai, China) [39]. Raw sequencing reads were processed on the Majorbio Cloud Platform.

### Isolation and identification of *Clostridium* strains

Rhizosphere slurries from iron plaque-treated microcosms were serially diluted in sterile, anoxic phosphate buffer (20 mM, pH 6.0). For plating, 100 μl of each dilution was spread onto ferric citrate–glucose agar plates (1.5% (w/v) agar). This medium consisted of a nitrogen-free Hoagland solution supplemented with 56 mM ferric citrate and 20 mM glucose (pH 6.0) [40]. Following 5–7 days of anoxic incubation at 30°C, colonies exhibiting distinct and consistent morphology (opaque, circular, convex) were isolated. Isolates were repeatedly streaked onto fresh ferric citrate–glucose agar to obtain pure cultures, which were subsequently verified by 16S rRNA gene sequencing.

Genomic DNA was extracted from a pure isolate using the FastDNA Spin Kit for Soil (MP Biomedicals) according to the manufacturer’s instructions. The genome was sequenced using a NovaSeq 6000 System (paired-end 2 × 150 bp; Illumina, USA) and PacBio Sequel IIe (10 kb, two SMRT cells). Sequencing reads were quality-trimmed using fastp v0.23.0, and the clean short reads and HiFi reads were assembled with Flye v2.9.2 and polished using Pilon v1.22, followed by quality assessment with N50. Protein-coding sequences (CDSs) were predicted using Prodigal v2.6.3 and annotated with BLAST and DIAMOND tools against the NR and KEGG databases (*e*-value ≤1e^−5^). The 16S rRNA gene of isolate genome shared 99.7% sequence identity and 100% query coverage with *Clostridium saccharoperbutylacetonicum* strain N1-4 (HMT) (accession CP004121.1). Consequently, the isolate was identified as *C. saccharoperbutylacetonicum* strain RS-1, and its genome is available in NCBI GenBank under BioProject PRJNA1395845 (accession CM137652.1).

Bacterial morphology of the isolated *C. saccharoperbutylacetonicum* RS-1 was examined by a transmission electron microscope (TEM, HT7700, Hitachi, Japan). Cells were grown to mid-log phase in the standard nitrogen-free Hoagland medium supplemented with 20 mM glucose under anoxic conditions at 30°C. Cells were then harvested, fixed with 2.5% glutaraldehyde, negatively stained with 1% phosphotungstic acid (pH 7.0), and observed under an accelerating voltage of 80 kV.

### Quantification of Fe(II), glucose, metabolites, riboflavin, ATP, and NAD(P)H

Fe(II) and glucose were measured using the ferrozine method and the 3,5-dinitrosalicylic acid method, respectively, as described previously [41, 42]. Major fermentation products, including acetate, butyrate, ethanol, and butanol, were analyzed by gas chromatography (GC; 7890, Agilent, USA) and confirmed by gas chromatography–mass spectrometry (GC–MS; 8890-7000E, Agilent, USA). Other acidic metabolites including lactate, pyruvate, and formate were quantified using high-performance liquid chromatography (HPLC; LC-2030C, Shimadzu, Japan), and acetone was determined by GC (GC9720PLUS, Fuli Instruments, China). Gaseous products (CO_2_ and H_2_) were analyzed with a gas chromatograph (GC-14, Shimadzu, Japan), and dissolved inorganic carbon (IC) was measured with a total organic carbon analyzer (TOC-L CPH, Shimadzu, Japan). IC concentrations were converted to CO_2_ equivalents and combined with headspace CO_2_ to obtain total CO_2_ yield. Biomass, expressed as C_5_H_7_O_2_N stoichiometry, was calculated from cell dry weight [37]. The major metabolic products from steady-state fermentation are summarized in Supplementary Table S2.

Riboflavin was quantified using a fluorescence spectrophotometer (G9800A, Agilent, USA) by collecting excitation spectra from 300 to 500 nm with the emission wavelength fixed at 525 nm [43]. Intracellular ATP levels were determined using the Enhanced ATP Assay Kit (S0027, Beyotime, China) [44]. The NAD(H)/NAD^+^ and NADP(H)/NADP^+^ ratios were determined using the NAD(H) Quantification Kit (MAK037, Sigma-Aldrich, USA) and the NADP(H) Assay Kit (BC1105, Solarbio, China), respectively [44, 45].

### Metabolomics and metabolic flux analysis

Untargeted metabolomics was conducted to compare the metabolic profiles of *C. saccharoperbutylacetonicum* RS-1 under conditions with and without iron plaque reduction. Briefly, whole-cell cultures were harvested at steady state, lysed by ultrasonication, filtered through a 0.22 μm membrane, frozen in liquid nitrogen, and stored at −80°C until analysis. Metabolite separation was performed on a UHPLC-Orbitrap Exploris 480 system (Thermo Fisher Scientific, USA) equipped with an ACQUITY HSS T3 column (100 × 2.1 mm, 1.8 μm; Waters, USA). To monitor the stability of the analysis throughout the process, a pooled quality control (QC) sample, prepared by mixing equal volumes of all analytical samples, was injected at regular intervals. Mass spectrometry data were acquired in both positive and negative ionization modes over a mass range of m/z 70–1050. Raw data were processed with Progenesis QI (Waters Corporation, USA) for feature detection, alignment, and normalization. Metabolites were identified by matching accurate mass and MS/MS spectra against the HMDB and Metlin databases. Statistical and bioinformatic analyses, including multivariate analysis and pathway enrichment, were performed on the Majorbio Cloud Platform. Twelve biological replicates were analyzed per group. The resulting metabolomic profiles are provided in Supplementary Data S1.

For metabolic flux analysis, major products quantified at steady state were used. To enable direct comparison, all quantities (including gases and biomass) were converted to molar amounts (mol) based on measured liquid and headspace volumes. These values were then normalized to the moles of glucose consumed and are expressed on a per-mole-of-glucose basis (mol mol^−1^). Biomass formation was represented using the empirical formula C_5_H_7_O_2_N. To reduce model complexity, a lumped hexose-phosphate pool detected in untargeted metabolomics was used as a stoichiometric proxy for unmetabolized hexose and other unquantified upstream intermediates, as previously reported [46]. Carbon conversion efficiency (CCE), defined as the ratio of carbon recovered in metabolic products to the total carbon input from glucose [47], was calculated as Equation (1):


1 \begin{eqnarray*}
\mathrm{CCE}=\sum(C_{\mathrm{metabolite}}\times n_{\mathrm{C}})/(C_{\mathrm{glucose}\ \mathrm{consumed}} \times 6)\times 100
\end{eqnarray*}

where *C*_metabolite_ represents the amount of each quantified metabolic product (mol), including organic acids, solvents, gases (CO_2_), and biomass; *n*_C_ represents the number of carbon atoms per molecule of a metabolite; *C*_glucose consumed_ represents the amount of glucose consumed (mol).

Glucose oxidation efficiency represents the fraction of the theoretical maximum electrons released from glucose oxidation (24 mol e^−^/mol) [48], calculated as Equation (2):


2 \begin{eqnarray*}\text{Glucose oxidation efficiency}=&\ \sum(C_{\mathrm{metabolite}}\times N_{\text{electron released}})/\\&(C_{\text{glucose consumed}}\times24)\times100\% \end{eqnarray*}

where *N*_electron released_ represents electrons released per mole of metabolite (mol e^−^/mol). The calculations of glucose oxidation efficiency are provided in Supplementary Table S3.

### Transcriptome sequencing and analysis

Transcriptomes of strain RS-1 grown under iron plaque-reducing conditions and under plaque-free conditions were sequenced and analyzed. Briefly, cells were harvested at the mid-log phase, flash-frozen in liquid nitrogen, and stored at −80°C until RNA extraction. Total RNA was extracted using TRIzol reagent (Invitrogen, USA). Ribosomal RNA was depleted with the RiboCop rRNA Depletion Kit for Mixed Bacterial Samples (Lexogen, USA). Strand-specific RNA-seq libraries were constructed using the Illumina Stranded RNA Prep (Ligation) kit and sequenced on a NovaSeq 6000 System (2 × 150 bp; Illumina, USA).

For transcript quantification, reads were aligned to the newly assembled genome of strain RS-1. Gene expression levels were reported as fragments per kilobase per million mapped reads (FPKM) using RSEM. For differential expression analysis, raw read counts were analyzed with DESeq2, with significance defined as |log_2_(fold change)| ≥ 1 and an adjusted *P* < .05 (Benjamini-Hochberg method). The complete transcriptomic dataset is provided in Supplementary Data S2.

### Metagenome sequencing and analysis

Genomic DNA was sheared to ~350 bp fragments using a Covaris M220 (Gene Company Limited, China). A paired-end sequencing library was constructed with the NEXTFLEX Rapid DNA-Seq Kit (Bioo Scientific, USA) and sequenced on a NovaSeq X Plus System (Illumina) using NovaSeq X Series 25B Reagent Kits. Raw reads were quality-filtered using fastp (v0.23.0) to retain high-quality reads (Q ≥ 20, length ≥ 50 bp). These reads were de novo assembled with MEGAHIT (v1.2.9). Contigs ≥500 bp were used for open reading frame (ORF) prediction via Prodigal (v2.6.3), and predicted ORFs ≥100 bp were retained. A non-redundant gene catalog was generated from these ORFs using CD-HIT (v4.6.1; parameters: 90% identity, 90% coverage). Representative sequences from this catalog were functionally annotated against the KEGG database using DIAMOND (v2.0.13; *e*-value ≤1e-5). Gene abundance in each sample was estimated by mapping reads back to the catalog using SOAPaligner (v2.21). To identify *Clostridium* species affiliated with key respiratory genes, sequences annotated with the KOs for *apbE* (K03734), *nosR* (K19339), *cydA* (K00425), and *cydB* (K00426) were extracted and taxonomically classified using the NCBI NR database. The metagenomic data are available on the NCBl Sequence Read Archive under accession PRJNA1443245 and details regarding the identified *Clostridium* species are provided in Supplementary Data S3.

## Results

### Iron plaque reduction enhances BNF of *Clostridium* in the rice rhizosphere

**Figure 2:**
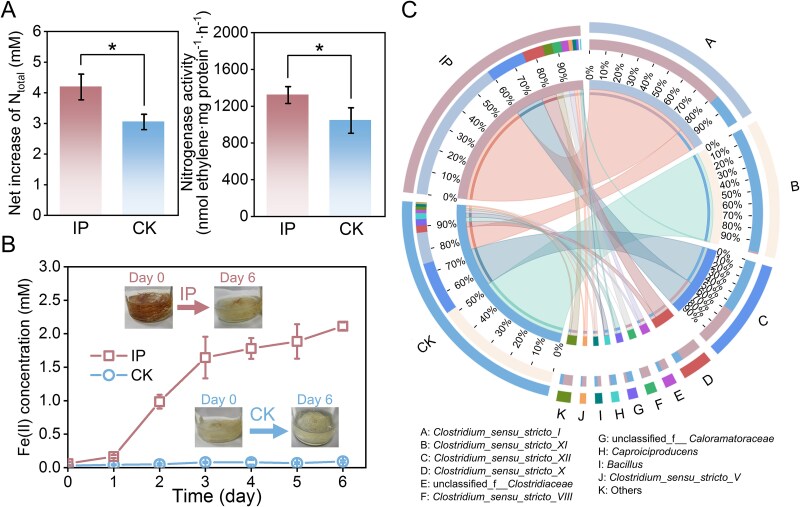
The impact of iron plaque on nitrogen fixation, iron reduction, and microbial community composition in rice root microcosms. (a) Net increase in total nitrogen (*N*_total_, left panel) and nitrogenase activity (right panel) in rhizosphere microcosms with (IP) and without (CK) iron plaque. Net increase of N_total_ was calculated as the difference in *N*_total_ between day 0 and day 6 of incubation. Data are presented as mean ± standard deviation for three biologically independent replicates. Statistical significance was determined by a two-tailed Student’s *t*-test (^*^P < .05). (b) Fe(II) concentration in the culture medium over a 6-day incubation period. Insets show representative images of the root at day 0 and day 6, illustrating its visual transformation. (c) Comparative taxonomic composition at the genus level, visualized using Circos plots, for communities with and without iron plaque. On the left, two outer arcs of equal length represent the total community in the IP and CK groups, respectively. On the right, the 11 dominant genera are shown as outer arcs arranged from top to bottom in decreasing overall relative abundance; arc length is proportional to each genus’s combined relative abundance across both groups (IP + CK), and colors denote different genera. The inner ring on the right indicates the fraction of each genus contributed by IP versus CK (i.e. the within-genus partitioning between groups), whereas the inner ring on the left shows the relative abundance of each of the 11 genera within the IP and CK communities (i.e. the within-group composition). The results showed the presence of iron plaque significantly increases the relative abundance of *Clostridium sensu stricto I* but does not substantially restructure the overall community composition.

In our primary attempts to study the effect of iron plaque on the BNF in the rice rhizosphere, we established a microcosm and measured nitrogen accumulation. Over 6 days, the presence of iron plaque resulted in a net increase in total nitrogen (*N*_total_) of 4.19 ± 0.42 mM, which is ~1.4-fold higher than in its absence (3.05 ± 0.25 mM; Fig. 2a). Consistent with this trend, nitrogenase activity, determined via the acetylene reduction assay, was ~1.3 times higher in the presence of iron plaque (1322.16 ± 91.66 vs. 1044.24 ± 138.94 nmol C_2_H_4_ mg protein^−1^ h^−1^). Concurrently, the iron plaque was largely dissolved by day 6, with the concentration of Fe(II) in the culture medium increasing from undetectable levels to 2.11 ± 0.06 mM (Figs 2b and S1), suggesting the reduction of iron plaque. Together, these results indicate iron plaque reduction contributes to the enhancement of BNF in the rice rhizosphere.

We analyzed the microbial community by 16S rRNA gene sequencing. *Clostridiaceae* was the dominant family in the rhizosphere (Supplementary Fig. S2). In the presence of iron plaque, the relative abundance of genus *Clostridium sensu stricto I, Clostridium sensu stricto VIII*, and *Clostridium sensu stricto X* increased significantly (Fig. 2c), with *Clostridium sensu stricto I* showing the most pronounced rise, from 12.3% to 59.2% (Fig. 2c). In contrast, other major *Clostridium* groups including *Clostridium sensu stricto XI* and *Clostridium sensu stricto XII*, decreased in relative abundance, falling from 29.7% to18.2%, and from 45.7% to 0.5%, respectively, in the absence of iron plaque. *Clostridiaceae* have been reported to be as one of the dominant microbial communities in the flooded, iron-rich paddy soil [19, 20, 49], and frequently encode nitrogen-fixing genes [23]. Even though they typically perform fermentative growth, recent studies indicated that some of *Clostridium* could reduce Fe(III) [47, 50]. The selective enrichment of specific *Clostridium* lineages under iron plaque and nitrogen-deficient conditions thus suggests that Fe(III) reduction may benefit these taxa during BNF.

**Figure 3:**
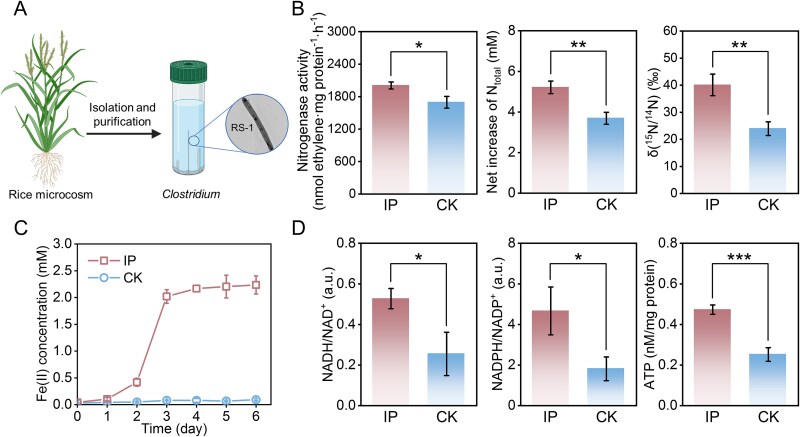
Isolation and characterization of *Clostridium* with and without iron plaque. (a) Isolation of the *C. saccharoperbutylacetonicum* strain RS-1 from an iron plaque microcosm. (b) Net increase in nitrogenase activity (left panel), total nitrogen (middle panel), and the ^15^N/^14^N isotope ratio (right panel) in cultures with (IP) and without (CK) iron plaque. (c) Fe(II) concentration in the culture medium over a 6-day incubation. Strain RS-1 could reduce the iron plaque. (d) Intracellular NADH/NAD^+^ ratio (left panel), NADPH/NADP^+^ ratio (middle panel), and ATP concentration (right panel) with and without iron plaque. The presence of iron plaque elevates both reducing power (NADH and NADPH) and cellular energy (ATP). Data are presented as mean ± standard deviation for three biologically independent replicates. Statistical significance was determined by a two-tailed Student’s *t*-test (^*^P < .05, ^**^P < .01, ^***^P < .001).

We attempted to isolate the *Clostridium* species from the iron plaque-treated microcosm for subsequent characterization. The isolate, identified as *C. saccharoperbutylacetonicum* strain RS-1 (a member of *Clostridium* sensu stricto I), carries nitrogen-fixation genes (Figs 3a and S3). We examined its BNF in the presence of iron plaque. It exhibited higher nitrogenase activity (2006.19 ± 64.89 nmol C_2_H_4_ mg protein^−1^ h^−1^) compared to cultures without iron plaque (1695.49 ± 108.12 nmol C_2_H_4_ mg protein^−1^ h^−1^) (Fig. 3b, left). Consistently, total nitrogen accumulation reached 5.21 ± 0.31 mM after 6 days in the presence of plaque (Fig. 3c, middle). ^15^N-isotope labeling confirmed enhanced BNF, with a δ^15^N value of 40.12 ± 3.98‰ in iron plaque-amended cultures, ~1.7-fold higher than in controls (23.95 ± 2.53‰; Fig. 3b, right). Concurrently, the concentration of Fe(II) in the medium increased significantly, reaching 2.24 ± 0.17 mM by day 6. These results demonstrate that iron plaque reduction stimulates BNF in *C. saccharoperbutylacetonicum* RS-1.

### The fermentation of *Clostridium* was reprogramed via reducing iron plaque

*C. saccharoperbutylacetonicum* is known for anaerobic fermentation [51], our isolated strain RS-1 also reduces extracellular Fe(III) oxides during this process (Fig. 3c). Because fermentative Fe(III) reduction may facilitate intracellular redox homeostasis and influence fermentation profiles, we assessed the intracellular redox status of strain RS-1 during iron plaque reduction. Compared with cells without iron plaque treatment, RS-1 cells performing iron plaque reduction showed 2.12- and 2.57-fold increases in the NADH/NAD^+^ and NADPH/NADP^+^ ratios, respectively (Fig. 3d, left and middle). In parallel, intracellular ATP increased to 0.47 ± 0.02 nmol (mg protein)^−1^, nearly twice that under conditions without Fe(III) plaque (0.25 ± 0.03 nmol (mg protein)^−1^) (Fig. 3d, right). This elevated reducing power and ATP production could support the energy-intensive process of nitrogen fixation. However, because iron plaque reduction is expected to consume reducing equivalents, the observed accumulation of NAD(P)H is unexpected.

**Figure 4:**
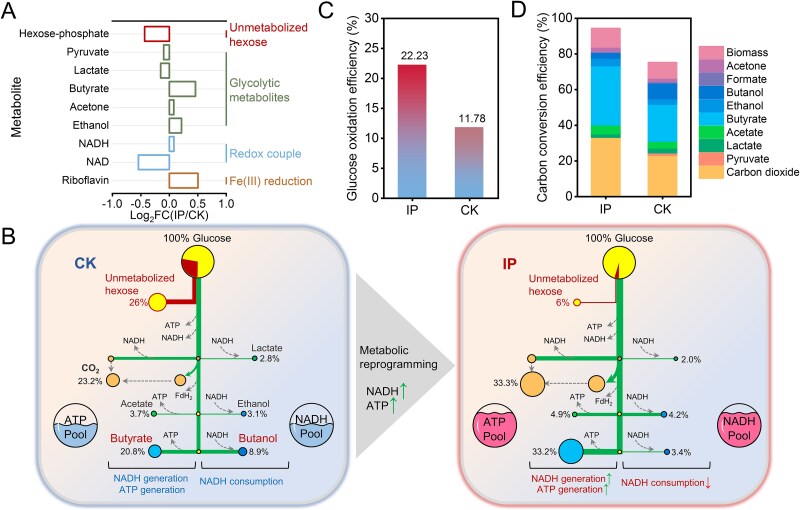
Comparative metabolic analysis of *Clostridium saccharoperbutylacetonicum* with and without iron plaque. (a) Differential metabolites (expressed as log2 fold change) in cultures with iron plaque (IP) compared to those without (CK). Data are presented as mean ± standard deviation for three biologically independent replicates. Statistical significance was determined by a two-tailed Student’s *t*-test. (b) A schematic overview of metabolic pathways without (left panel) and with (right panel) iron plaque, highlighting glucose utilization, unmetabolized hexose accumulation, and ATP/NADH generation. Percentages shown below metabolites indicate the carbon conversion efficiency (CCE) of each component relative to glucose consumption, calculated from measured metabolic product profiles (Supplementary Table S2). Hexose was used as a stoichiometric proxy for unmeasured metabolites and determined via mass balance (Supplementary Table S4). The presence of iron plaque induced a metabolic shift from solventogenesis to acidogenesis, increasing the cellular ATP and NADH pools. (c) Glucose oxidation efficiency (%) in the presence and absence of iron plaque. (d) Carbon conversion efficiency (CCE, %) and the distribution of end-products. Iron plaque redirects carbon flux toward acidogenic products.

To identify the basis of the increased reducing power, we performed comparative metabolomics under conditions with and without iron plaque reduction (Fig. 4a and Supplementary Data S1). Iron plaque reduction induced a metabolic shift in strain RS-1 (Supplementary Fig. S4), with the most pronounced changes occurring in carbohydrate metabolism and amino acid (Supplementary Fig. S5). This is consistent with altered carbon fermentation and nitrogen conversion during plaque reduction. We therefore focused on fermentation-related pathways. The major fermentation end products were similar in both conditions, including lactate, butyrate, acetone, and ethanol. However, several unmetabolized intermediates such as hexose phosphate and pyruvate decreased during iron plaque reduction, suggesting more complete carbon flux through fermentation (Fig. 4a). In addition, NADH and NAD^+^ exhibited a positive and negative log_2_ fold change, respectively, in agreement with the increased NADH/NAD^+^ ratio observed during iron plaque reduction (Fig. 3d). Because the fermentation of glucose to pyruvate consumes NAD^+^ to generate NADH, the elevated flux likely contributed to the observed NADH accumulation.

We quantified glucose fermentation using HPLC, GC, and colorimetric methods (Supplementary Table S2; a summary is shown in Fig. 4b, with complete data in Supplementary Fig. S6). The stoichiometry of the two fermentation pathways was verified by calculating both mass balances and electron equivalent balances (Supplementary Tables S4 and S5) [52]. Consistent with the metabolomics data, acidogenesis predominated in the presence of Fe(III) plaque, with butyrate accounting for 33.2% of carbon products compared to 20.8% in its absence (Fig. 4b). Acetate showed a similar increase (4.9% with Fe(III) plaque vs. 3.7% without Fe(III) plaque) (Fig. 4b). In contrast, solventogenesis was strongly suppressed, as indicated by the reduced butanol yield in the presence of Fe(III) plaque (3.4%) relative to its absence (8.9%) (Fig. 4b). In addition, both CO_2_ and biomass represented larger fractions of the carbon output when Fe(III) plaque was present. Accordingly, glucose oxidation efficiency (the fraction of the theoretical maximum electrons released from glucose oxidation) (Fig. 4c) and carbon conversion efficiency (CCE; the fraction of consumed glucose carbon entering energy-producing pathways) (Fig. 4d) increased to 22.2% and ~94.3%, respectively, with Fe(III) plaque, compared to 11.8% and ~75.2% without Fe(III) plaque. Because glucose oxidation efficiency and CCE collectively enhance ATP and NADH generation, these results indicate that Fe(III) plaque reduction promotes more complete glucose oxidation by partially shifting fermentation from solventogenesis toward acidogenesis, which likely contributes to enhanced nitrogen fixation.

### Coupling iron plaque reduction with BNF in *Clostridium*

**Figure 5:**
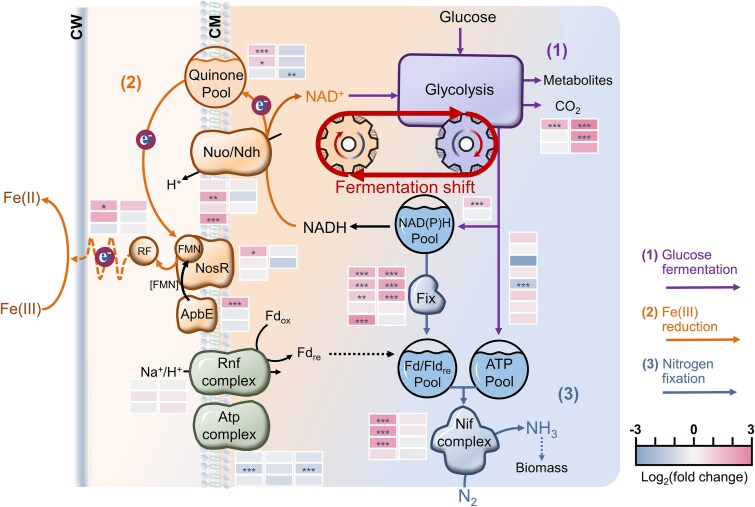
Proposed mechanism for fermentative iron reduction coupled with nitrogen fixation in *Clostridium saccharoperbutylacetonicum*. Comparative transcriptomic profiles of key genes involved in glucose fermentation (violet), iron reduction (orange), and nitrogen fixation (blue) in cultures with versus without Fe(III) plaque were shown. Data are presented as mean ± standard deviation for three biologically independent replicates. Statistical significance was determined by a two-tailed Student’s *t*-test (^*^P < .05, ^**^P < .01, ^***^P < .001). Iron(III) reduction drives the NAD^+^ regeneration. It shifts the metabolic balance from solventogenesis toward the more energy-efficient acidogenic pathway, increasing ATP and reducing power (NADH/NADPH) to support nitrogen fixation. CM, cell membrane; CW, cell wall; fix, electron bifurcation complex (oxidizes NAD(P)H to generate reduced ferredoxin (Fd_red_) or flavodoxin (Fld_red_)); Nif, nitrogenase complex; Nuo/Ndh, proton-translocating NADH: Quinone oxidoreductase; Rnf, proton-translocating ferredoxin: NAD^+^ oxidoreductase (uses reverse electron transport to drive ferredoxin reduction); ApbE, flavinyl transferase; RF, riboflavin; FMN, flavin mononucleotide; NosR, electron delivery protein containing FMN-binding domains. The proposed electron transfer pathway initiates with NADH oxidation by Nuo/Ndh, reducing quinone. Reduced quinone subsequently reduces NosR, a protein flavinylated by ApbE. Electrons are then channeled from NosR to riboflavin, culminating in iron plaque reduction.

*Clostridium* are Gram-positive bacteria and are generally considered inefficient at EET [53]. To determine how *Clostridium* seeks iron plaque reduction to alter intracellular fermentation, we compared the transcriptomic profiles of cells grown under conditions with and without iron plaque reduction. The most affected genes were related with catalytic activity, binding, cellular anatomical structure, and cellular processes (Supplementary Fig. S7). Correspondingly, carbohydrate metabolism, amino acid metabolism, and energy metabolism were actively affected (Supplementary Figs S8 and S9), which is consistent with the metabolomics data. In particular, genes involved in glucose fermentation for acidogenesis and in nitrogen fixation were upregulated, indicating synergy between energy production and nitrogen fixation (Fig. 5).

Extracellular iron(III) oxide reduction typically functions as a form of anaerobic respiration that can contribute to oxidative phosphorylation. In our study, genes encoding NADH-quinone oxidoreductase, which catalyzes NADH oxidation to reduce quinone as part of the electron transport chain, were significantly upregulated (Fig. 5). In contrast, ATP synthase genes were downregulated (Fig. 5). This expression pattern suggests that Fe(III) reduction in this system does not primarily support ATP synthesis, but instead helps maintain redox balance by regenerating NAD^+^. Nevertheless, NADH oxidation still contributes to generating a transmembrane proton gradient. Genes for the Rnf complex were also upregulated (Fig. 5). This complex is known to harness the proton gradient while oxidizing NADH to reduce ferredoxin [54]. Thus, rather than being used for ATP production, the proton gradient appears to be consumed by the Rnf complex to produce reduced ferredoxin, which in turn supplies electrons to support nitrogen fixation.

**Figure 6:**
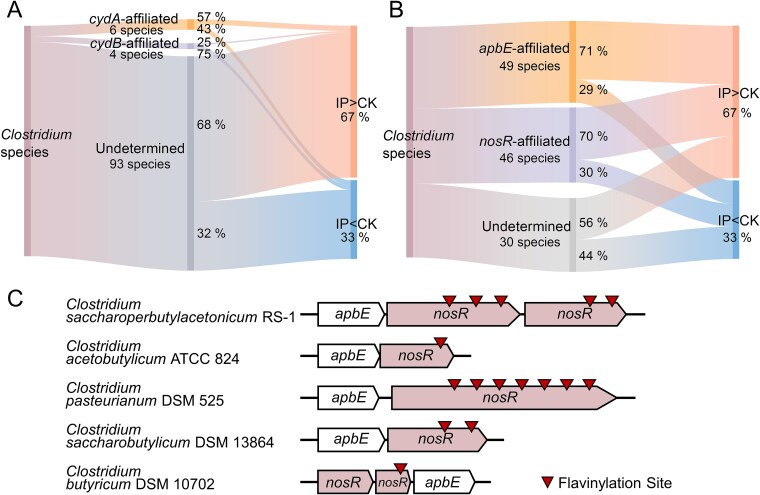
Genetic distribution of ApbE-NosR electron transfer module in *Clostridium*. (a) Sankey diagram showing the distribution of *Clostridium* species affiliated with *cyd* genes in microcosms with iron plaque (IP) versus a no-plaque control (CK). (b) Sankey diagram showing the distribution of *Clostridium* species affiliated with the *apbE* and *nosR* genes under the same conditions. The “undetermined” category includes taxa where specific genes were absent from metagenomic data, potentially due to low abundance, copy number, or true genetic absence. (c) Conserved genomic organization of the *apbE-nosR* module across different iron-reducing *Clostridium* species. Inverted triangles denote conserved, periplasm-facing FMN-binding motifs. This conserved architecture suggests a shared genetic basis for directing electron transfer from the membrane to the periplasm.

Microbes typically employ cytochrome-based EET pathways to reduce extracellular iron(III) oxides [55]. Although two inner membrane cytochromes are predicted in strain RS-1, only the gene for the Cyd cytochrome *bd* ubiquinol oxidase was significantly upregulated (Supplementary Data S2). Cyd can oxidize the reduced quinone pool, potentially shuttling electrons to other carriers [56]. However, metagenomic analysis revealed that most iron plaque-enriched *Clostridium* species lack *cyd* (Fig. 6a). Furthermore, Cyd has been shown to attenuate EET in the Gram-positive bacterium *Enterococcus faecalis* [57]. Taken together, these findings suggest that Cyd is not a universal EET mechanism for iron(III) oxide reduction in *Clostridium*.

Unlike cytochrome-dependent pathways, many Gram-positive bacteria employ a flavinylation-based system for EET [58, 59]. The genome of strain RS-1 encodes the core components of such a system: a membrane protein with multiple FMN-binding domains (NosR) and the flavinyl transferase ApbE, which is essential for protein flavinylation [60]. Both corresponding genes were significantly upregulated during iron plaque reduction and are predominantly found in iron plaque-enriched *Clostridium* species (Figs 5 and 6B), suggesting a conserved role for iron plaque reduction. NosR is proposed to receive electrons from the quinone pool for extracellular transfer [61]. We infer that it could thereby facilitate Fe(III) reduction. Supporting this, *Clostridium intestinale*, which lacks the NosR-ApbE system, cannot reduce extracellular iron(III) oxide (Supplementary Fig. S10). Conversely, all reported iron(III)-reducing *Clostridium* species (*C. acetobutylicum* [25], *C. pasteurianum* [26], *C. butyricum* [27], and *C. saccharobutylicum* [28]) possess the NosR-ApbE system but not the Cyd (Fig. 6c). Therefore, we propose that the NosR-ApbE system, rather than Cyd, is responsible for extracellular iron(III) oxide reduction in strain RS-1.

Strain RS-1 reduced iron plaque even when physically separated from it by a semipermeable membrane (3 kDa cutoff), indicating a capacity for mediated Fe(III) reduction (Supplementary Fig. S11). Riboflavin is a redox-active molecule known to mediate EET in Gram-positive bacteria, including *C. acetobutylicum* [25]. In strain RS-1, genes related with riboflavin production were significantly increased during iron-plaque reduction (Fig. 5), and a substantial amount was detected in the culture medium (ca. 50 nM) (Supplementary Fig. S11). Given its established role in shuttling electrons across the cell envelope in other systems, we propose that secreted riboflavin acts as an electron shuttle, facilitating the reduction of extracellular iron(III) oxide by strain RS-1 (Fig. 5).

## Discussion

Our study reveals a rhizosphere mechanism in which iron plaques act as a reactive Fe(III) reservoir and redox interface. By serving as a terminal electron acceptor, they shift fermentative *Clostridium* metabolism toward high-energy-yield acidogenesis, increasing ATP availability and thereby enhancing the energy-intensive BNF in rice rhizosphere. This metabolic reprogramming is facilitated by a speculated flavinylation-based EET pathway that channels electrons to extracellular Fe(III). Glycolysis continuously generates NADH, which must be reoxidized to NAD^+^ to sustain metabolic flux [24]. *Clostridium* typically addresses this through solventogenic pathways such as acetone–butanol–ethanol fermentation, which consume reductants but yield no additional ATP [62], creating a critical trade-off between redox balance and energy conservation. Our findings demonstrate that electron transfer to Fe(III) plaques alleviates the necessity for NADH dissipation through solventogenesis. This relaxes intracellular redox pressure. However, unlike previous reports showing the reduction of NADH accumulation [25, 63], our results show the accumulation of NAD(P)H as carbon flux partially redirects toward acidogenic pathways [64, 65]. Therefore, the Fe(III)-coupled metabolic reprogramming effectively decouples redox balancing from low-energy-yield metabolism, simultaneously boosting the availability of both ATP and reductants, two prerequisites for nitrogenase activity.

The enhancement of BNF by fermentative iron(III) oxide reduction broadens the conceptual landscape of Fe–N coupling in paddy soils. Iron plaque, which is rich in reactive Fe, forms at the oxic–anoxic boundary in paddies. It creates a dynamic oxygen microenvironment which has been thought to favor microbial nitrification and nitrogen fixation [66]. In addition, iron plaque can directly influence the nitrogen transformations through Fe redox reactions. For example, Fe(II) oxidation can be coupled to denitrification [36], whereas Fe(III) respiratory reduction can be coupled to ammonium oxidation [67] or linked to methanotrophic nitrogen fixation [68]. The coupling of clostridial nitrogen fixation with Fe(III) reduction has been predicted a long time ago [18, 69]. Our study provides mechanistic details and reveals a new paradigm that Fe(III) reduction shifts the fermentative physiology of fermentative diazotrophs into a regime more favorable for sustained nitrogen fixation. In this framework, Fe(III) reduction is not a respiratory process. Rather, it provides an additional electron disposal reaction that supports continued acidogenesis. This activity drives the accumulation of NAD(P)H, increases ATP yield per unit substrate, and maintains redox cycling via extracellular electron disposal.

The enrichment of specific *Clostridium* species under our Fe(III)-reducing conditions indicates that iron plaques can act as deterministic selectors within the rhizosphere diazotrophic community. It should be noted, however, that this does not imply *Clostridium* would dominate the natural rice rhizosphere microbiome. Indeed, preliminary global metagenomic analysis confirms that canonical Fe(III)-respiring diazotrophs, such as *Geobacter* and *Anaeromyxobacter*, are the dominant taxa in natural environments (Supplementary Fig. S12 and Supplementary Data S4). This discrepancy likely stems from our specific experimental conditions: we supplied glucose as the sole electron donor, whereas *Geobacter* and *Anaeromyxobacter* typically utilize low-molecular-weight organic acids. Nevertheless, in the natural rhizosphere, these dominant diazotrophs still need to depend on fermentation products generated by populations like *Clostridium* to drive nitrogen fixation, ultimately contributing to a robust and cooperative diazotrophic consortium. Hence, the mechanistic insights gained from *Clostridium* may inform future studies on the metabolic flexibility of other diazotrophs in various anoxic environments, like wetlands or lake sediments. Our experimental conditions also featured stable, preprocessed iron plaques, which differ from the heterogeneous and redox-fluctuating plaques in natural rhizospheres. Future work should investigate how such dynamic oscillations affect the metabolic shift observed in *Clostridium*.

To date, research on EET mechanisms of *Clostridium* species remain in their infancy [53, 63]. *Clostridia* generally lack cytochromes and the related electron transfer pathway [70]. Exogeneous redox mediators, such as neutral red and methyl viologen could mediate the electron uptake of *Clostridium* from the cathode [71]. A recent study showed that *Clostridium* recruits the EET pathway of an electroactive partner to enable iron(III) oxide reduction [53]. Our metabolomic, transcriptomic, metagenomic, and comparative genomic analyses collectively support a model for a flavin-based EET pathway. In this model, a flavinylated NosR protein oxidizes the quinone pool and transfers electrons to the periplasm, where riboflavin further shuttles them to facilitate Fe(III) reduction. Follow-up tests, such as gene knockouts where feasible, or chemical inhibition with appropriate controls will be important to validate the process. Establishing this causality would not only strengthen the mechanistic claim but also clarify whether plaque-coupled BNF can be predicted from genomic markers of mineral interaction.

From an applied perspective, the work suggests that managing the availability of rhizosphere electron acceptors, specifically the formation and persistence of Fe(III) plaque, could provide an effective way to support biological N inputs. Although iron plaques are already known to enhance phosphorus uptake and mitigate heavy-metal toxicity in rice [72], our findings indicate that strategies which maintain appropriate redox oscillations and promote plaque formation such as via controlled water management or iron supplementation could further stimulate diazotrophic activity by *Clostridium* and similar populations. This offers a potential pathway to improve the energetic efficiency of rhizosphere nitrogen fixation and reduce dependence on synthetic fertilizers. However, any such management approach must be evaluated within the context of whole-ecosystem trade-offs, including its effects on methane emissions, nitrous oxide dynamics, and the mobilization of nutrients and metal(loids) at the root interface.

## Supplementary Material

wrag088_Supplemental_Files

## Data Availability

All data generated or analyzed during this study are included in this published article.
