# Supplementary material for: Iron plaques as terminal electron acceptors optimize clostridial fermentation and nitrogen fixation in rice rhizospheres
Source: ISME J. 2026 Apr 14;20(1):wrag088. doi: 10.1093/ismejo/wrag088 (PMC13134045; doi:10.1093/ismejo/wrag088)
Supplement: wrag088_Supplemental_Files [file wrag088_supplemental_files.zip › The_Supplementary_Material_04.06_wrag088.pdf]

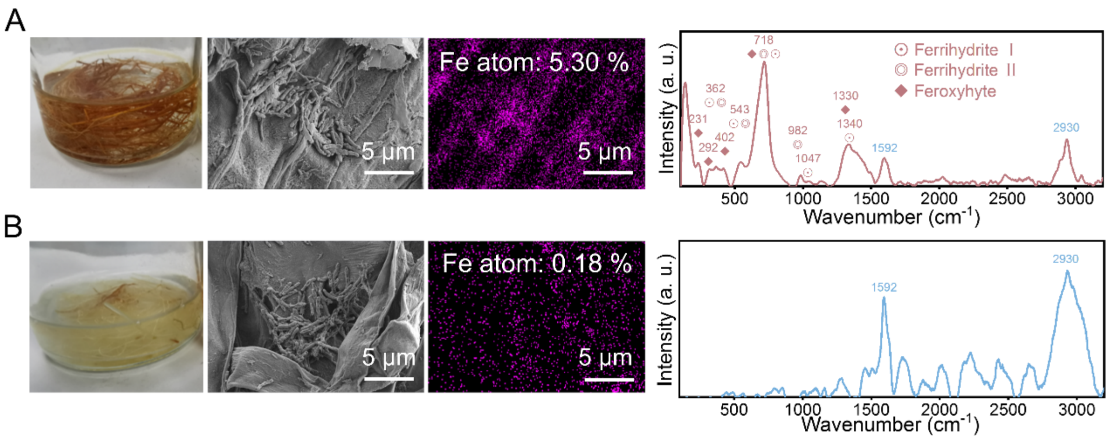

Figure S1. Characterization of iron plaque reduction in rice rhizosphere microcosms at Day 0 (A) and Day 6 (B). Representative photographs (left) show a root-surface color change from reddish-brown (Fe(III)-dominated) to nearly colorless (dissolved Fe(II)) after 6 days. SEM–EDS (middle; SU8010, Hitachi, Japan) revealed a marked decrease in root-surface Fe atomic percentage. Confocal Raman spectroscopy (right; inVia, Renishaw, UK; 532 nm) shows the characteristic broad bands corresponding to ferrihydrite, confirming the presence of iron plaque [1]. The disappearance of ferrihydrite signatures on the rice roots by day 6 suggests the dissolution of iron plaque. For SEM, roots were fixed in 2.5% glutaraldehyde (5 h), dehydrated in graded ethanol (25, 50%, 75%, 90%, and 100%; 15 min each), freeze-dried (1 day), and gold-sputter coated (Emitech K550X, UK).

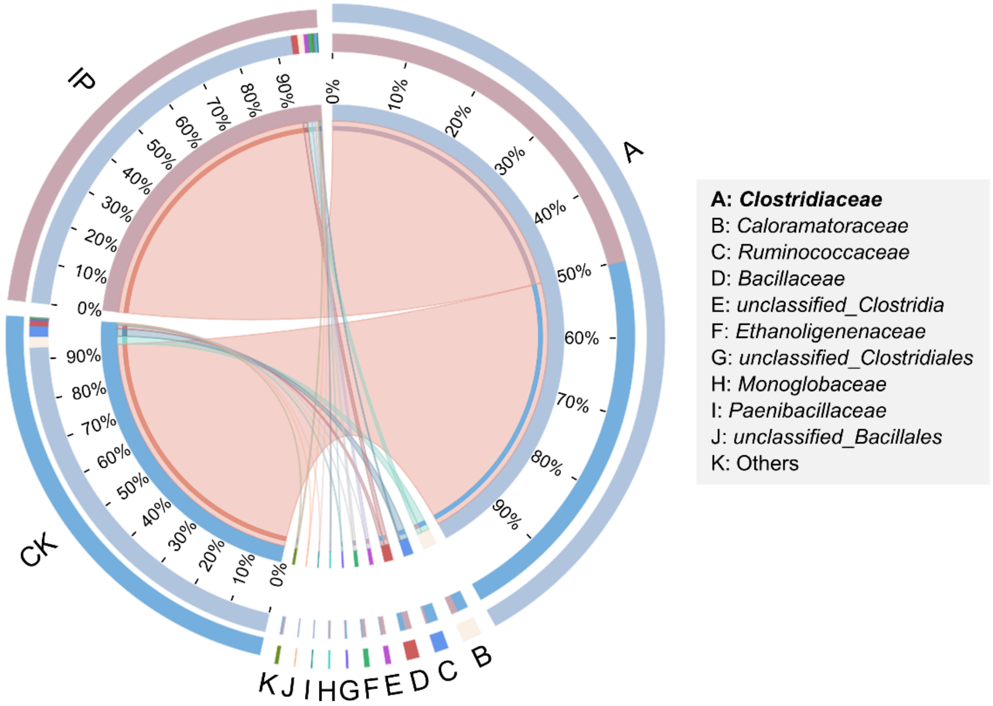

Figure S2. Circos plots showing comparative taxonomic composition at the family level of microbial communities in rice root microcosms with and without iron plaque. *Clostridiaceae* was the dominant family in the rhizosphere.

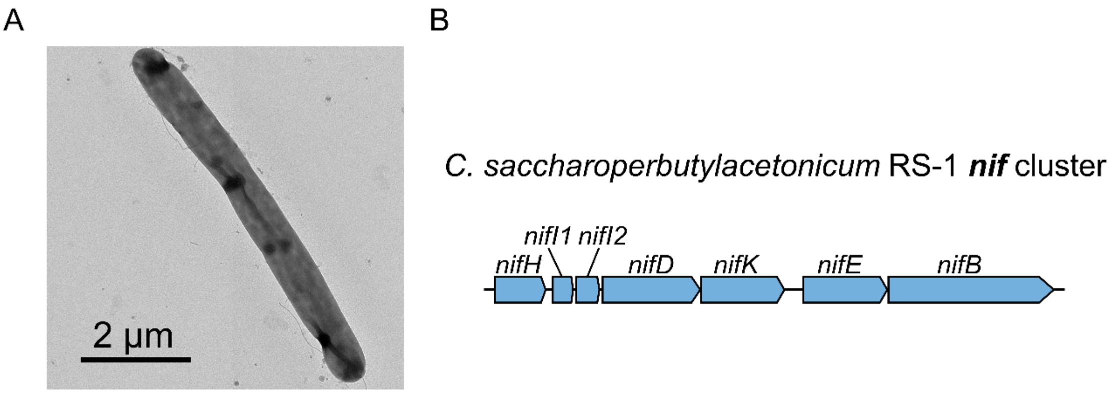

Figure S3. Morphological characterization and nitrogenase gene cluster of the isolate *C. saccharoperbutylacetonicum* RS-1. (A) Transmission electron micrograph (TEM) of the isolated *C. saccharoperbutylacetonicum* RS-1. **(B)** Schematic organization of the nitrogenase (*nif*) gene cluster in the isolate. Arrows indicate predicted open reading frames and their transcriptional orientation.

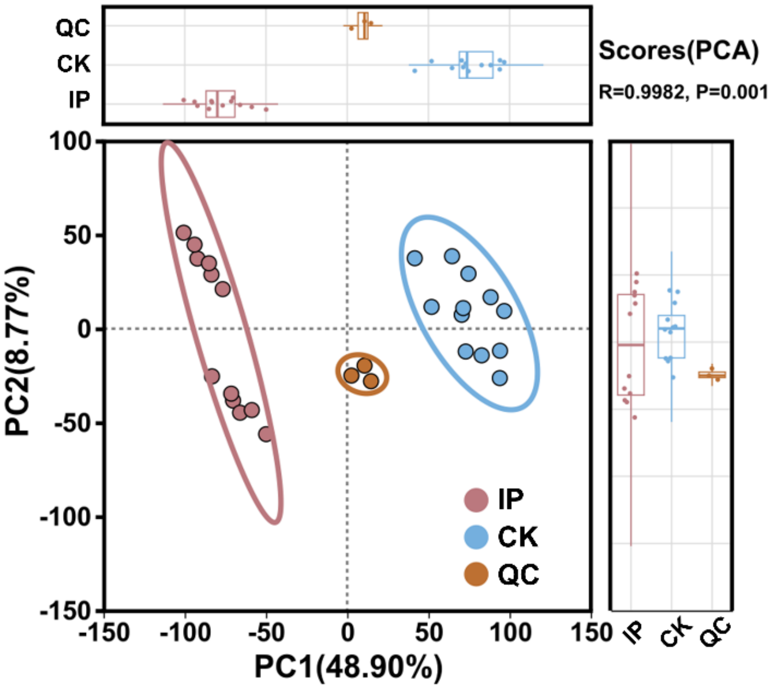

Figure S4. Principal component analysis (PCA) of untargeted metabolomics of *Clostridium saccharoperbutylacetonicum* strain RS-1 grown with iron plaque (IP) and without iron plaque (CK). Each treatment included 12 biological replicates. A pooled quality-control (QC) sample, prepared by combining equal volumes of all samples, was analyzed to monitor analytical stability.

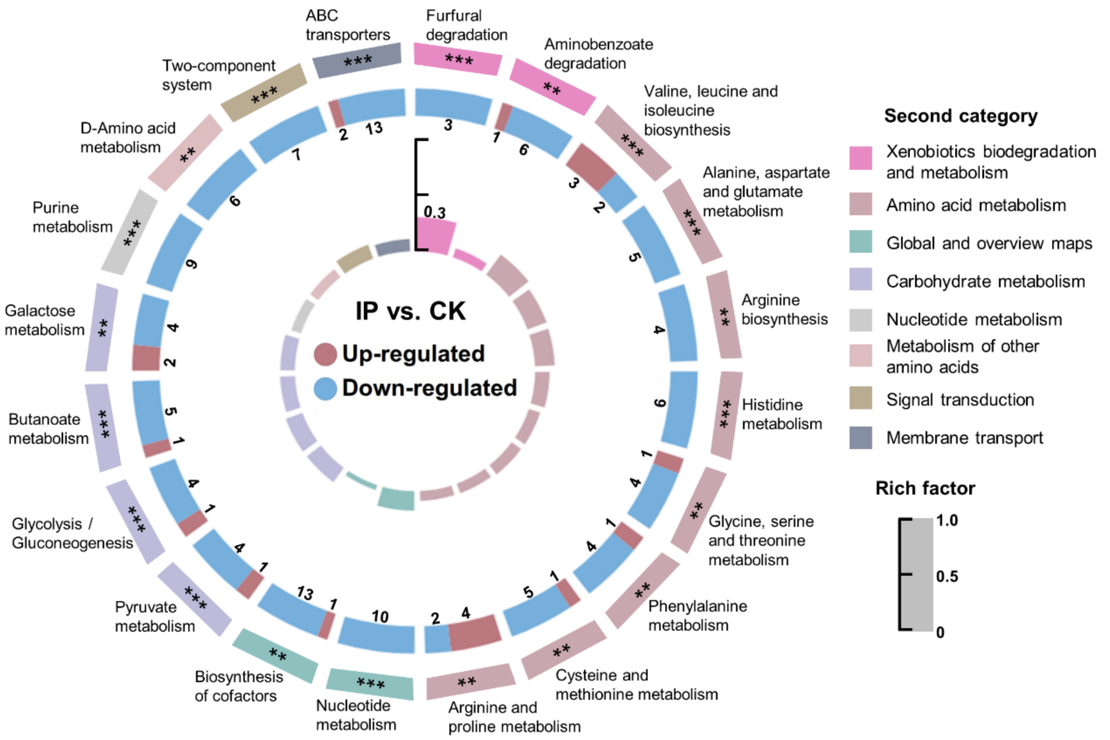

Figure S5. Multi-pathway enrichment analysis based on comparative metabolomics of *C. saccharoperbutylacetonicum* RS-1 under conditions with and without iron plaque, showing the metabolic shift induced by iron plaque. **p < 0.01, ***p < 0.001.

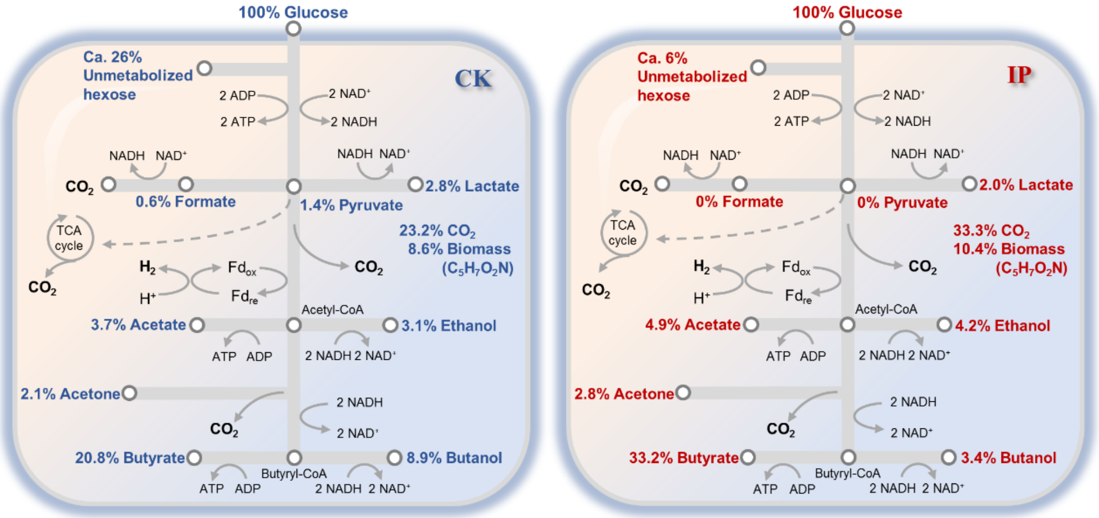

Figure S6. Schematic overview of metabolic pathways without (left panel) and with (right panel) iron plaque. The complete metabolic profiling data (based on Table S2), which details the distribution of glucose-derived products, reveal that iron plaque induces a metabolic shift from solventogenesis to acidogenesis and enhances glucose oxidation, thereby increasing ATP and NADH production for nitrogen fixation.

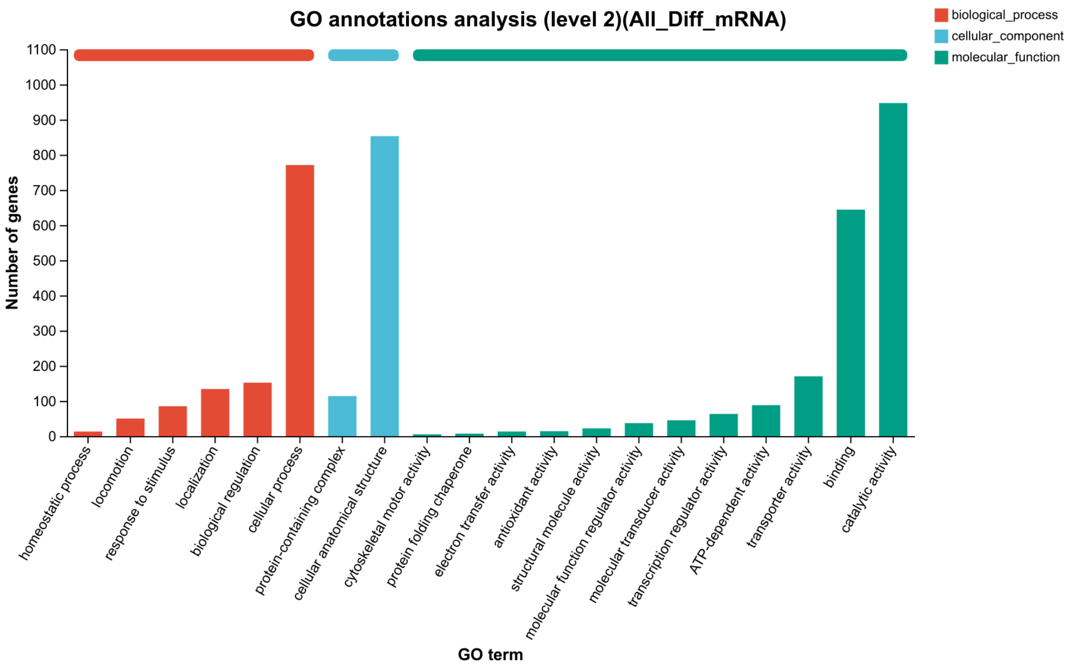

Figure S7. GO (level 2) classification of all differentially expressed genes (DEGs; All_Diff_mRNA) in *C. saccharoperbutylacetonicum* RS-1 under conditions with and without iron plaque. Bars indicate the number of DEGs assigned to GO level-2 terms within biological process (red), cellular component (blue), and molecular function (green), with major categories including cellular process, cellular anatomical structure, binding and catalytic activity.

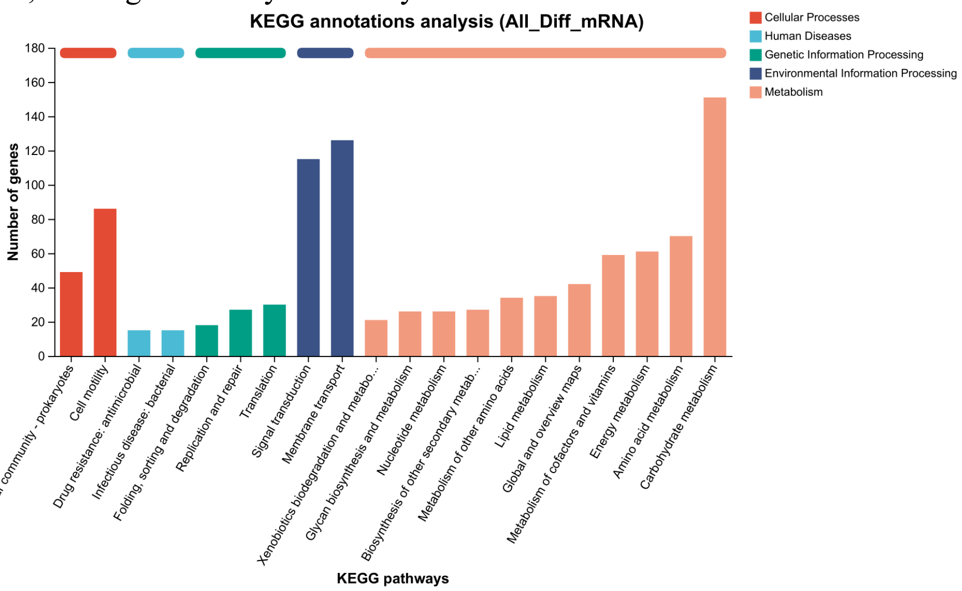

Figure S8. KEGG functional classification of all differentially expressed genes (DEGs; All_Diff_mRNA) in *C. saccharoperbutylacetonicum* RS-1 under conditions with and without iron plaque. Bars indicate the number of DEGs assigned to KEGG pathway categories, grouped into Cellular Processes (red), Human Diseases (light blue), Genetic Information Processing (green), Environmental Information Processing (dark blue), and Metabolism (orange). Major categories included cell motility, signal transduction, membrane transport, energy metabolism, amino acid metabolism and carbohydrate metabolism.

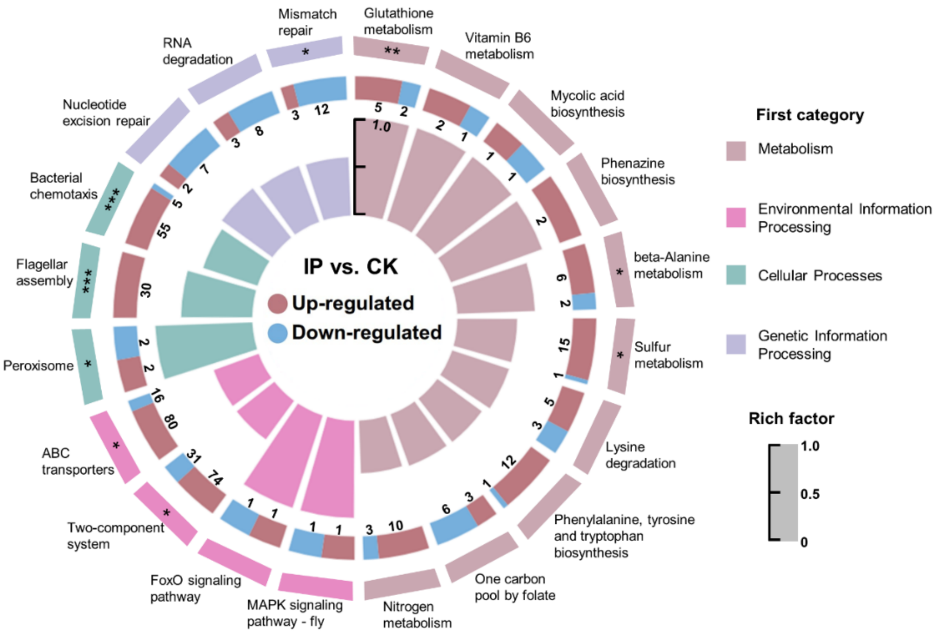

Figure S9. Multi-pathway enrichment analysis based on transcriptome of *C. saccharoperbutylacetonicum* RS-1 under conditions with and without iron plaque reduction, revealing the transcriptional mechanisms underlying the iron plaque-induced metabolic shift. *$p < 0.05$, **$p < 0.01$, ***$p < 0.001$.

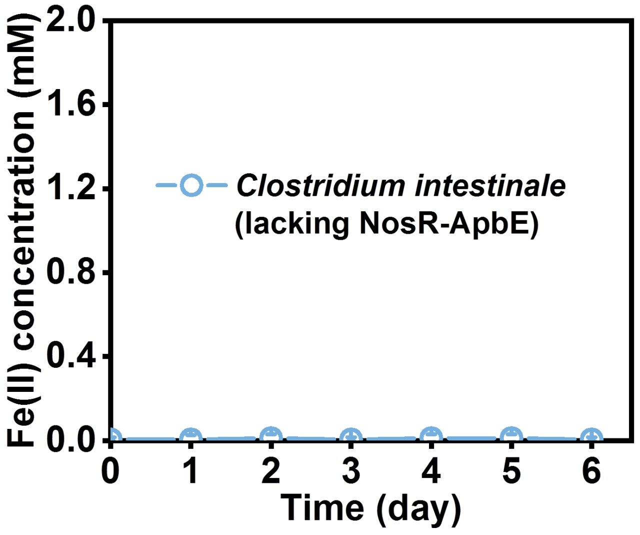

Figure S10. Extracellular Fe(III) oxide reduction assay of *Clostridium intestinale* Lx1, showing that the strain, which lacks the NosR-ApbE system, is incapable of reducing extracellular iron(III) oxide. *Clostridium intestinale* was routinely cultured in ACE fermentation medium under anaerobic, dark conditions at 30 °C. The ACE medium was composed of the following components (per liter): NH_4Cl 1.00 g, KH_2PO_4 0.33 g, K_2HPO_4 0.45 g, $MgSO_4 \cdot 7H_2O$ 0.10 g, D-fructose 10.00 g, yeast extract 2.00 g, $NaHCO_3$ 10.00 g, L-cysteine-HCl·H_2O 0.50 g, $Na_2S \cdot 9H_2O$ 0.50 g, DL mineral solution 10.00 mL, and DL vitamin solution 10.00 mL.

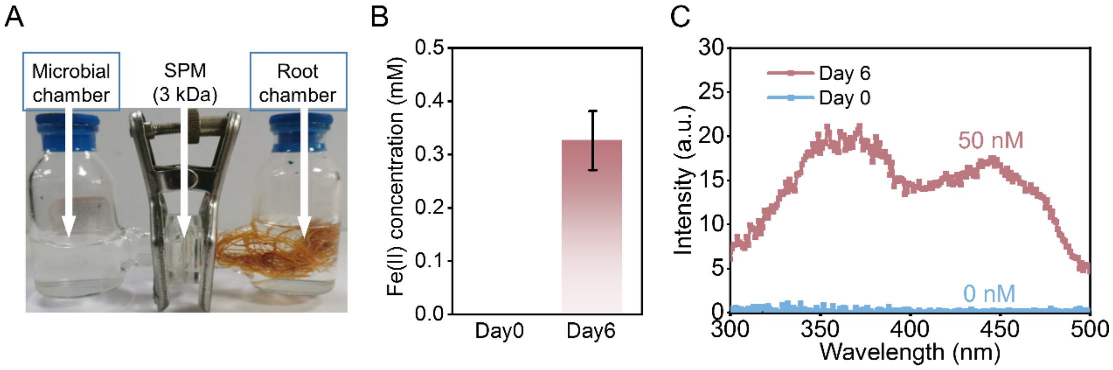

Figure S11. Two-chamber (H-type) separation assay demonstrating the indirect extracellular Fe(III) reduction capacity of *Clostridium saccharoperbutylacetonicum* RS-1. (A) Schematic of an H-type dual-compartment reactor, separated by a 3 kDa semi-permeable membrane (SPM). *C. saccharoperbutylacetonicum* RS-1 was inoculated into the microbial chamber, while rice roots bearing iron plaque were placed in the root chamber, and 20 mM glucose was supplied as the substrate. **(B)** Increase in Fe(II) concentration in the root chamber over 6 days of anaerobic incubation, indicating reduction of root-associated Fe(III) plaque mediated by diffusible electron shuttles (e.g., riboflavin). **(C)** Fluorescence excitation spectra of samples from the root chamber, confirming the presence and quantifying the concentration of riboflavin. For measurement, filtered culture medium (3 mL) were analyzed using a fluorescence spectrophotometer (G9800A, Agilent, USA) by recording excitation spectra from 300 to 500 nm with emission fixed at 525 nm; the characteristic excitation maxima at 370 nm and 445 nm confirmed the presence of riboflavin [2]. The absence of bacterial contamination in the root chamber was confirmed by PCR (data not shown).

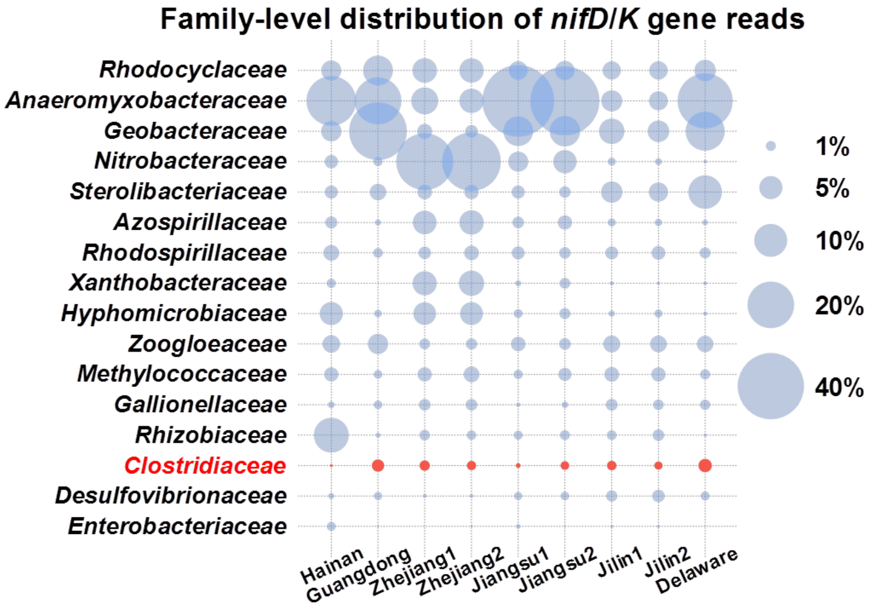

Figure S12. Family-level distribution of *nifD* and *nifK* genes in publicly available metagenomic datasets from six paddy fields across China and the United States, showing that *Clostridiaceae* was one of the dominant diazotrophic taxa in the rhizosphere. Sequences annotated with nitrogenase genes *nifK* (K02586) and *nifD* (K02591) were extracted following a previously described approach [1]. All datasets were obtained from NCBI and processed on the Majorbio Cloud Platform. Details of metagenomic datasets used are provided in Supplementary Data S4.

Table S1. The physicochemical properties of the paddy soil.

**Parameter**	**Content**
[a]Moisture content (%)	24 ± 2
[b]Nitrate nitrogen (mg/kg)	6.63 ± 0.20
[c]Nitrite nitrogen (mg/kg)	0.08 ± 0.02
[d]Ammonia nitrogen (mg/kg)	18.79 ± 0.32
[e]Total nitrogen (mg/kg)	30.30 ± 0.53
[f]Dissolved organic carbon (mg/kg)	31.32 ± 0.38
[g]Inorganic carbon (mg/kg)	1.83 ± 0.26
[h]Total carbon (mg/kg)	33.15 ± 0.46
[i]Ferrous iron (g/kg)	0.18 ± 0.02
[j]Total iron (g/kg)	11.88 ± 0.42

Note: [a]Moisture content was determined gravimetrically by oven-drying [2]. [b]Nitrate (NO_3^-), [c]nitrite (NO_2^-) and [d]ammonium (NH_4^+) were quantified by dual-wavelength spectrophotometry, the N-(1-naphthyl) ethylenediamine dihydrochloride colorimetric method and the indophenol blue method, respectively [3]. [e]Total nitrogen was determined by persulfate digestion (Hach kits 2672245, USA). [f]Dissolved organic carbon (DOC) and [g]inorganic carbon (IC) were measured after filtration of extracts through a 0.45 μm membrane using a TOC analyzer (TOC-L CPH, Shimadzu, Japan); [h]total carbon was calculated as DOC + IC. [i]Ferrous iron was extracted with HCl and quantified by the ferrozine assay at 562 nm [3]. [j]Total iron was extracted with HCl plus hydroxylamine hydrochloride and quantified using the same ferrozine assay.

Table S2. Metabolic product profiles of Fe(III)-coupled and classic fermentation at steady state.

	Compound	Formula	Fe(III)-coupled fermentation	Fermentation
**Consumed substrate (mmol/L)**	Glucose	$C_6H_{12}O_6$	26.42 ± 0.33	26.43 ± 0.22
**Main fermentative products (mmol/L)**	Pyruvate	$C_3H_4O_3$	0 ± 0	0.75 ± 0.20
	Lactate	$C_3H_6O_3$	1.04 ± 0.06	1.45 ± 0.28
	Acetate	$C_2H_4O_2$	3.9 ± 0.29	2.91 ± 0.55
	Butyrate	$C_4H_8O_2$	13.16 ± 2.48	8.24 ± 2.90
	Ethanol	C_2H_6O	3.34 ± 0.24	2.48 ± 0.31
	Butanol	$C_4H_{10}O$	1.36 ± 0.43	3.53 ± 0.32
	Formate	CH_2O_2	0 ± 0	0.91 ± 0.03
	Acetone	C_3H_6O	1.47 ± 0.43	1.12 ± 0.33
	[a]Carbon dioxide	CO_2	52.77 ± 2.88	36.75 ± 0.83
	Hydrogen	H_2	47.13 ± 3.32	25.64 ± 7.48
**Other products (mmol/L)**	Ferrous	Fe^{2+}	2.24 ± 0.17	-
	[b]Total nitrogen	-	5.21 ± 0.31	3.69 ± 0.30
	Biomass	$C_5H_7O_2N$	3.30 ± 0.41	2.73 ± 0.18
**Nitrogen fixation efficiency (mol N/mol comsumed glucose)**			**0.20**	**0.14**

[a]Including gaseous carbon dioxide and dissolved inorganic carbon.

[b]Including nitrogen in biomass, ammonium, and other organic forms.

Table S3. Glucose oxidation efficiencies of Fe(III)-coupled and classic fermentation.

**Compound**	Glucose	Pyruvate	Lactate	Acetate	Butyrate	Ethanol	Butanol	Formate	Acetone	Carbon dioxide	**Glucose oxidation efficiency (%)**
**Formula**	$C_6H_{12}O_6$	$C_3H_4O_3$	$C_3H_6O_3$	$C_2H_4O_2$	$C_4H_8O_2$	C_2H_6O	$C_4H_{10}O$	CH_2O_2	C_3H_6O	CO_2	
**Released electron equivalents (mol e^-/mol product formation)**	-	2	0	0	-4	-4	-8	2	-4	4	
	[a]Reactants (mol)	[a]Products (mol)									
**IP**	1	0.00	0.04	0.15	0.5	0.13	0.05	0.00	0.06	1.98	
**CK**	1	0.03	0.05	0.11	0.31	0.09	0.13	0.03	0.04	1.39	
	Maximum releasable electron equivalents (mol e^-)	Released electron equivalents (mol e^-)									
**IP**	24.0	0.00	0.00	0.00	-1.99	-0.51	-0.41	0.00	-0.23	7.99	**20.23**
**CK**	24.0	0.06	0.00	0.00	-1.25	-0.38	-1.07	0.07	-0.17	5.56	**11.78**

[a]Normalized C-containing metabolites relative to glucose.

Table S4. Mass balances of Fe(III)-coupled and classic fermentation.

**Compound**	Glucose	Dinitrogen	Ferrihydrite	Proton	Phosphate	Pyruvate	Lactate	Acetate	Butyrate	Ethanol	Butanol	Formate	Acetone	Carbon dioxide	Hydrogen	Ferrous	Biomass	Other organic N	Water	Proton	Hexose-phosphate
**Formula**	$C_6H_{12}O_6$	N_2	$Fe(OH)_3$	H^+	PO_4^{3-}	$C_3H_4O_3$	$C_3H_6O_3$	$C_2H_4O_2$	$C_4H_8O_2$	C_2H_6O	$C_4H_{10}O$	CH_2O_2	C_3H_6O	CO_2	H_2	Fe^{2+}	$C_5H_7O_2N$	NH_4^+	H_2O	H^+	$C_6H_{13}O_9P$
	[a]Reactants (mol)					[a]Products (mol)															
**IP**	1	0.1	0.08	[c]0	[c]0.06	0.00	0.04	0.15	0.5	0.13	0.05	0.00	0.06	1.98	1.78	0.08	0.12	[c]0.08	[c]0.08	[c]0.10	[bc]0.06
C			6											6							
H			12.24											12.24							
O			6.48											6.48							
Fe			0.08											0.08							
N			0.2											0.2							
P			0.06											0.06							
**CK**	1	0.07	0	[c]0.3	[c]0.26	0.03	0.05	0.11	0.31	0.09	0.13	0.03	0.04	1.39	0.97	0.00	0.10	[c]0.04	[c]0.32	[c]0.00	[bc]0.26
C			6											6							
H			12.3											12.3							
O			7.04											7.04							
Fe			0											0							
N			0.14											0.14							
P			0.26											0.26							

[a]Normalized data relative to glucose.

[b]Hexose-phosphate was employed to compensate for unmeasured residual metabolites as a stoichiometric proxy.

[c]Calculated.

Table S5. Electron equivalent balances of Fe(III)-coupled and classic fermentation.

Compound	Glucose	Dinitrogen	Ferrihydrite	Proton	Phosphate	Pyruvate	Lactate	Acetate	Butyrate	Ethanol	Butanol	Formate	Acetone	Carbon dioxide	Hydrogen	Ferrous	Biomass	Other organic N	Water	Proton	Hexose-phosphate	Electron equivalent balance (%)
**Formula**	$C_6H_{12}O_6$	N_2	$Fe(OH)_3$	H^+	PO_4^{3-}	$C_3H_4O_3$	$C_3H_6O_3$	$C_2H_4O_2$	$C_4H_8O_2$	C_2H_6O	$C_4H_{10}O$	CH_2O_2	C_3H_6O	CO_2	H_2	Fe^{2+}	$C_5H_7O_2N$	NH_4+	H_2O	H^+	$C_6H_{13}O_9P$	
**Electron equivalent (mol e⁻/mol compound)**	24	0	0	0	0	10	12	8	20	12	24	2	16	0	2	1	20	3	0	0	24	
			Reactants (mol)									Products (mol)										
**IP**	1	0.1	0.08	0	0.06	0.00	0.04	0.15	0.5	0.13	0.05	0.00	0.06	1.98	1.78	0.08	0.12	0.08	0.08	0.10	0.06	
**CK**	1	0.07	0	0.3	0.26	0.03	0.05	0.11	0.31	0.09	0.13	0.03	0.04	1.39	0.97	0.00	0.10	0.04	0.32	0.00	0.26	
			Electron equivalents of reactants (mol e^-)									Electron equivalents of products (mol e^-)										
**IP**	24	0	0	0	0	0	0.48	1.2	10	1.56	1.2	0	0.96	0	3.56	0.08	2.4	0.24	0	0	1.44	**96.33**
**CK**	24	0	0	0	0	0.3	0.6	0.88	6.2	1.08	3.12	0.06	0.64	0	1.94	0	2	0.12	0	0	6.24	**96.58**

Data S1. (Separate file)
Differential metabolites of *Clostridium saccharoperbutylacetonicum* RS-1 under Fe(III)-coupled fermentation and classic fermentation

Data S2. (Separate file)
Differential transcriptome of *Clostridium saccharoperbutylacetonicum* RS-1 under Fe(III)-coupled fermentation and classic fermentation

Data S3. (Separate file)
Analysis of candidate Fe(III)-reduction genes in *Clostridium* from microcosms with and without iron plaque.

Data S4. (Separate file)
Metagenomic datasets for profiling diazotrophic *Clostridiaceae* in the rice rhizosphere nitrogen-fixing community

References

1. Müller K, Ciminelli V S T, Dantas M S S, *et al.* A comparative study of As(III) and As(V) in aqueous solutions and adsorbed on iron oxy-hydroxides by Raman spectroscopy. *Water Res*. 2010;**44**:5660-72. https://doi.org/10.1016/j.watres.2010.05.053
2. Huang L, Tang J, Chen M, *et al.* Two modes of riboflavin-mediated extracellular electron transfer in *Geobacter uraniireducens*. *Front Microbiol*. 2018;**9**:2886. https://doi.org/10.3389/fmicb.2018.02886

1. Masuda Y, Mise K, Xu Z, *et al.* Global soil metagenomics reveals distribution and predominance of *Deltaproteobacteria* in nitrogen-fixing microbiome. *Microbiome*. 2024;**12**:95. https://doi.org/10.1186/s40168-024-01812-1
2. Anthony TL, Szutu DJ, Verfaillie JG, *et al.* Carbon-sink potential of continuous alfalfa agriculture lowered by short-term nitrous oxide emission events. *Nat Commun*. 2023;**14**:1926. https://doi.org/10.1038/s41467-023-37391-2
3. Ji Zhan, Lu Zhang, Shuyao Lai, *et al.* Fe(III)-dependent Nrf activity determines nitrate reduction partitioning in nitrate-reducing communities. *Mbio*. 2025;**16**:e02220-25. https://doi.org/10.1128/mbio.02220-25
4. McCarty PL. Thermodynamic electron equivalents model for bacterial yield prediction: modifications and comparative evaluations. *Biotechnol Bioeng*. 2007;**97**:377-88. https://doi.org/10.1002/bit.21250

5. Barin I. Thermochemical data of pure substances. Weinheim: VCH. 1989. https://doi.org/10.5860/choice.31-0632
6. Meurer F, Do HT, Sadowski G*, et al.* Standard Gibbs energy of metabolic reactions: II. Glucose-6-phosphatase reaction and ATP hydrolysis. *Biophys Chem*. 2017;**223**:30-8. https://doi.org/10.1016/j.bpc.2017.02.005
